# Re: A cross-sectional retrospective study comparing handwritten operation notes with electronic operation notes

**DOI:** 10.1308/rcsann.2023.0016

**Published:** 2023-04-13

**Authors:** WB Lo, JKK Chan, H Nishikawa

**Affiliations:** ^1^ Birmingham Children’s Hospital, UK; ^2^ Stoke Mandeville Hospital, Buckinghamshire Healthcare NHS Trust, UK

Ekowo *et al*'s article “A cross-sectional retrospective study comparing handwritten operation notes with electronic operation notes”,^1^ demonstrated better compliance of a good operation note by Royal College of Surgeons of England,^2^ providing compelling evidence for a complete shift to electronic format. We agree that we must progress into the 21^st^ century with electronic documentation but advocate caution. Completion of a prescribed operation note does not necessarily lead to a useful one.

Many surgeons rely on a diagrammatic representation to best relay their findings and steps taken during the surgery. In a recent survey by Kearns,^3^ 82% of surgeons reported that drawing helped communication with other professions and 80% agreed it helped communication with peers. However, at present, most electronic versions of operation notes do not provide adequate illustrative capabilities. We therefore wonder whether the electronic system at the authors’ institute has this, how many of the examined handwritten and electronic operation notes contained illustrations, and whether the prevalence was similar.

“A picture is worth a thousand words”; illustrations serve as “*aide mémoires*”, including recollection of the surgical pathology and procedure, and planning for further operations. Drawings are often considered clearer and more concise in describing (1) anatomical variations (e.g. gyral pattern of the brain in neurosurgery), (2) precise surgical interventions (e.g. creation of pedicled surgical flap in plastic surgery), and (3) relevant postoperative state (e.g. the remodelling of supraorbital bar and cranial vault in craniofacial surgery). Therefore, a comprehensive text operation note may not document the surgically most relevant information, which can be conveyed only by drawings.^4^ The primary sensory input during an operation is visual (and haptic) and, therefore, the most natural representation would also be visual. Documenting with drawings bypasses the two-stage translation of visual to verbal information by the surgeon, and from verbal back to visual again by the reader.

Procrastination of the surgical community to switch over wholesale from handwritten to electronic notes may in part be explained by surgeons’ preference to include a hand-drawn diagram.

We propose that ensuring good documentation of operations involves more than simply shifting to an electronic tick box system. It requires the foundations in place to enable this to happen, including the provision of the necessary illustrative software and hardware (e.g. stylus and tablet) packages and, while we are at it, for documentation of intraoperative photographs and scans. Such measures should lead to a step-change in the improvement of surgical documentation, reduce mistakes and improve care for our patients.

## References

[1] Ekowo O, Hammenga C, Altaf K *et al.* A cross-sectional retrospective study comparing handwritten operation notes with electronic operation notes. *Ann R Coll Surg Engl* 2023; **105**: 35–42. 35950972 10.1308/rcsann.2022.0066 PMC9773292

[2] Royal College of Surgeons of England. *The Royal College of Surgeons of England Good Surgical Practice*. RCS England. https://www.rcsed.ac.uk/media/414756/good-surgical-practice.pdf (cited January 2023).

[3] Kearns C. Is drawing a valuable skill in surgical practice? 100 surgeons weigh in. *J Vis Commun Med* 2019; **42**: 4–14. 30773071 10.1080/17453054.2018.1558996

[4] Sathi S, Rossitch E, Moore MR *et al.* Harvey Cushing’s postoperative sketches of pediatric brain tumors. *Child’s Nerv Syst* 1991; **7**: 56–58. 2054811 10.1007/BF00263836

